# Dialysis catheter fibrin sheath stripping: a useful technique after failed catheter exchange

**DOI:** 10.2349/biij.8.1.e8

**Published:** 2012-01-01

**Authors:** AF Mohamad Ali, E Uhwut, SK Liew

**Affiliations:** 1 Department of Radiology, Universiti Malaysia Sarawak, Kuching, Malaysia; 2 Department of Radiology, Sarawak General Hospital, Kuching, Malaysia

**Keywords:** Fine needle aspiration(FNA), pneumothorax, lung lesion, biopsy, CT

## Abstract

Fibrin sheath formation around long-term haemodialysis catheter is a common cause of failed dialysis access. Treatment options include pharmacological and mechanical methods. This paper reports a case of failed dialysis access due to fibrin sheath encasement. Pharmacologic thrombolysis, mechanical disruption using guide wire and catheter exchange had failed to address the issue. Eventually, fibrin sheath stripping using the loop snare technique was able to successfully restore the catheter function.

## INTRODUCTION

Tunnelled dual lumen catheters are frequently used for long-term haemodialysis access when arteriovenous fistulas or bypass grafts are maturing or failing. The commonest reason for failing long-term dialysis catheter is fibrin sheath formation. The term “fibrin sheath” is actually inaccurate because the sheath can be composed of thrombus, endothelial cells and collagen, depending on the duration of the catheter placement [1]. The sheath covers the inlet and outlet holes of haemodialysis catheters acting as a one-way valve. Even partial encasement can prevent high flow rates required for satisfactory haemodialysis [2]. Treatment options include pharmacological and/or mechanical methods. Pharmacological therapy involves instillation of urokinase (5,000 units or above) or tissue plasminogen activator (2.5 mg in 50 mls normal saline over 3 hours) to lyse the thrombus. Mechanical treatment includes catheter exchange, fibrin sheath disruption using guide wire and angioplasty balloon, as well as fibrin sheath stripping. This paper reports a case of fibrin sheath stripping after various methods (urokinase thrombolysis, wire disruption of fibrin sheath and catheter exchange) failed to restore catheter function.

## CASE REPORT

A 60 year-old Chinese male was referred to Sarawak General Hospital for failing dialysis access via his right permanent dual lumen haemodialysis catheter (PermCath, Quinton Instrument Co., Seattle, Washington, USA). The catheter was inserted 15 months ago and had been functioning well (baseline catheter flow > 300 ml/min) until recent months. A gradual decline in catheter flow from the arterial port was observed. Prior to the referral, 5,000 units of urokinase (Abbokinase, Abbott Laboratories, North Chicago, USA) was administered without much improvement (catheter flow < 150 ml/min).

**Figure 1:**
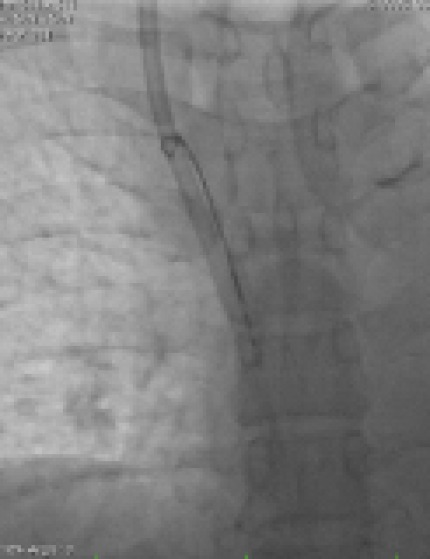
Fibrin sheath stripping using a Gunther Tulip IVC filter snare. The snare was tightened and pulled down along the shaft. Counter-traction was applied on the other end of the catheter to prevent dislodgement.

Ultrasound of neck revealed complete occlusion of both distal internal jugular veins and non-visualisation of both external jugular veins. Fluoroscopic screening showed no evidence of catheter tip malposition or kinking. Aspiration from the arterial port using a 20 cc Luer lock syringe (Medallion, Merit, Utah, USA) failed to yield any blood. Transcatheter venography via both lumens using non-ionic contrast media (Ultravist, Schering, Berlin, Germany) showed retrograde reflux of contrast along the catheter shaft, which is characteristic of fibrin sheath formation (Figure 1).

The catheter was successfully exchanged over a 0.035″ hydrophilic guide wire (Terumo, Japan). Persistent withdrawal occlusion (PWO) was still present after catheter exchange. A repeat venography showed persistent fibrin sheath surrounding the new catheter. An attempt to mechanically disrupt the fibrin sheath using the angled hydrophilic guide wire was only transiently effective. The patient was sent back to the ward for trial of haemodialysis on the following day, during which the flow rate was still persistently low (150 ml/min).

**Figure 2:**
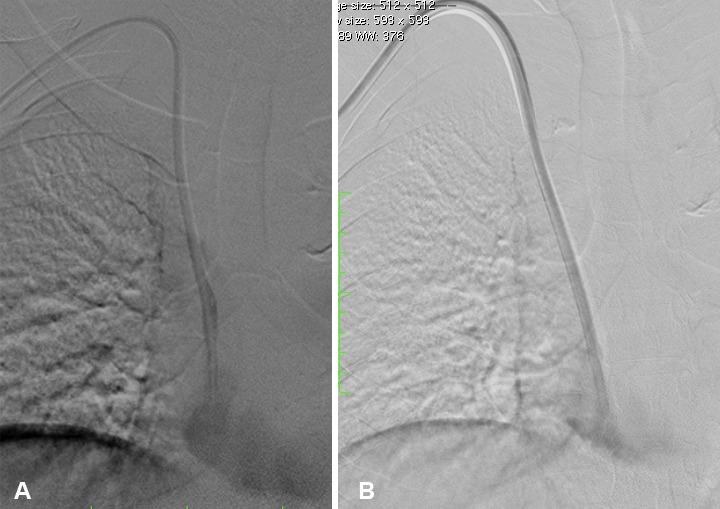
a) Contrast reflux along side the catheter shaft characteristic of fibrin sheath; b) After stripping, contrast reflux (fibrin sheath) is no longer demonstrated.

The decision to carry out fibrin sheath stripping was made after discussion with the attending nephrology team. PWO and catheter venography confirmed presence of the fibrin sheath. Right common femoral venous access was obtained. A Gunther Tulip Vena Cava Filter Retrieval set (Cook Medical, Bloomington, USA) was used. The snare was advanced as high up as possible around the catheter shaft in the superior vena cava. Several stripping passes were made by tightening the snare loop around the catheter while pulling the closed snare down the shaft. Gentle counter traction was applied by holding the port ends of the catheter to prevent catheter dislodgement. No fibrin tissue was noted on the snare loop upon withdrawal. Repeat venography showed rapid jet-flow from the catheter tip with resolution of the fibrin sheath (Figure 2). Close scrutiny of the pulmonary artery runs did not reveal any filling defects. Patient remained comfortable with no drop in oxygen saturation. Good flow via both ports were demonstrated. Subsequently, the patient underwent haemodialysis with improved catheter flow of 300 ml/min. The catheter flow remains good (> 300 ml/min) at 60 days follow up.

## DISCUSSION

Encasement of long-term haemodialysis catheter by fibrin sheath is a well-known complication, which interferes with the catheter function and prevents effective haemodialysis [3]. Fibrin sheath formation is seen in up to 76% of short- or long-term central venous catheter by pull-back venography [4]. In experimental studies, the fibrin sheath formation starts as early as 24 hours after insertion of the catheter, with encasement of its entire length within 5 to 7 days. The sheath begins as a thrombus containing some fibrin in the first few days and transforms to a cellular-collagen tissue after 1 week [1]. Upon catheter removal, the sheath tends to remain in the vein instead of attaching to the catheter [4].

In the case presented in this paper, the presence of fibrin sheath was confirmed on the initial venography. The local guidelines require catheter exchange (over a guide wire) as a solution to the problem. However, the fibrin sheath remained intact despite the catheter exchange, impairing the function of the newly-exchanged catheter. The acceptable option to salvage the new catheter was to strip the fibrin sheath. The fibrin sheath stripping was successfully performed using a Gunther Tulip IVC filter snare.

The only drawback of using the Gunther Tulip snare was its cost, which is equivalent to twice the price of the catheter itself. “Self-made” snare using exchange lengths of a suitable 0.018ʺ guide wire over an angled catheter, e.g. Kumpe, would offer a cheaper alternative. Re-puncture at a higher site on the bilateral internal jugular veins was not feasible as both veins were occluded. Embolisation to the lungs is likely as no fibrin tissue was seen on the snare loop; however, no adverse sequelae were observed during or immediately after the procedure.

## CONCLUSION

Stripping of the fibrin sheath is an option if catheter exchange fails to disrupt the persistent fibrin sheath. The only drawback is the high cost of using the IVC filter snare set. In the patient seen in this report, catheter patency remains good with flow above 300 ml/min at 60 days after the fibrin sheath stripping.

## References

[1] Forauer AR, Theoharis C (2003). Histologic changes in human vein wall adjacent to the indwelling central venous catheters. J Vasc Interv Radiol.

[2] Brady PS, Spence LD, Levitin A, Mickolich CT, Dolmatch BL (1999). Efficacy of percutaneous fibrin sheath stripping in restoring patency of tunneled haemodialysis catheters. AJR.

[3] Johnstone RD, Stewart GA, Akoh JA, Fleet M, Akyol M, Moss JG (1999). Percutaneous fibrin sheath stripping of failing dialysis catheter. Nephro Dial Transplant.

[4] Oguzkurt L, Tercan F, Torun D, Yildirim T, Zümrütdal A, Kizilkilic O (2004). Impact of short-term hemodialysis catheters on the central veins: a catheter venographic study. Eur J Radiol.

